# Nutritional Regulation of Poultry Meat Quality: Research Progress from Phenotypic Improvement to Mechanism Elucidation

**DOI:** 10.3390/foods15111986

**Published:** 2026-06-03

**Authors:** Lizhi Niu, Chenxu Wang, Ke Wu, Jinhua Cheng, Zhi Yang

**Affiliations:** 1 The Animal Science and Technology College, Yangzhou University, Yangzhou 255300, China; 17661386821@163.com (L.N.); 17706282279@163.com (C.W.); k1684242200@163.com (K.W.); 18118283131@163.com (J.C.); 2 Joint International Research Laboratory of Agriculture and Agri-Product Safety, Ministry of Education of China, Yangzhou University, Yangzhou 225009, China

**Keywords:** muscle fiber conversion, oxidative stability, gut–muscle axis, precision nutrition, functional feed additives

## Abstract

Poultry meat quality, as a key determinant of consumer acceptance and industrial economic efficiency, is shaped through the synergistic regulation of multiple factors including genetic background, husbandry management practices, and nutritional strategies. Among these, nutritional regulation has emerged as one of the most promising avenues for enhancing meat quality due to its high degree of operability and precision in intervention. This study systematically reviews the effects and mechanisms of nutritional strategies on poultry meat quality. These interventions entail modulating dietary protein and amino acids, fats and fatty acids, minerals and vitamins, alongside incorporating plant extracts and functional additives. It delves into the latest research advances in nutritional regulation within poultry meat quality. By dissecting the physiological and molecular mechanisms through which different nutritional strategies enhance meat quality, this study elucidates the scientific rationale for their effects via pathways regulating muscle metabolism, redox homeostasis, and microstructure. Accordingly, this review provides theoretical foundations and research directions for producing high-quality, high-value-added poultry meat products through precision nutritional strategies.

## 1. Introduction

Poultry meat occupies a dominant position in global meat consumption due to its high protein content, low fat levels, and efficient production. As consumer health awareness continues to grow, market demand has shifted from merely maximizing yield to pursuing superior meat quality, including color, water-holding capacity, tenderness, flavor, and nutritional composition [1,2]. Meat quality is a complex, multidimensional trait influenced by genetic background, rearing conditions, and nutritional management [3]. Among these factors, nutritional regulation is recognized as the most flexible, cost-effective, and precisely controllable intervention during commercial production cycles. Nutrients not only provide the material foundation for muscle tissue construction but also act as key signaling molecules that participate in regulating gene expression, metabolic pathways, and overall physiological status [4].

Over the past two decades, a substantial body of research has demonstrated that dietary manipulation can influence poultry meat quality through multiple nutrient categories. These include the strategic adjustment of crude protein levels and limiting amino acids, the supplementation of functional amino acids and their derivatives, the optimization of lipid sources and fatty acid profiles, the enrichment of vitamins and organic trace minerals, and the incorporation of plant extracts and probiotics [5,6,7,8,9]. Despite the diversity of these interventions, the reported effects converge on several core physiological pathways: modulation of muscle fiber type composition, enhancement of antioxidant defense systems and redox homeostasis, and regulation of gut microbiota.

However, several critical gaps remain unresolved in the current literature. First, most studies report phenotypic outcomes—such as shear force, drip loss, and color parameters—without adequately validating the underlying molecular mechanisms, leaving causal relationships uncertain. Second, interaction effects among different nutritional strategies are rarely investigated, despite the likelihood of synergistic or antagonistic interactions when multiple interventions are combined. Third, there is currently no integrative framework that links nutritional inputs to meat quality outcomes across multiple biological levels, from gene expression to tissue structure to sensory perception. Fourth, the translation of laboratory findings into commercial production systems remains challenging due to inconsistent results across breeds, rearing conditions, and baseline stress levels.

Therefore, this review summarizes recent progress in the nutritional regulation of poultry meat quality across five major nutrient categories—proteins and amino acids, lipids and fatty acids, vitamins and minerals, plant extracts, and probiotics. For each category, we examine the reported effects on meat quality traits and discuss the underlying physiological and molecular mechanisms where evidence permits. Specifically, we focus on three recurrent mechanistic themes in the literature: the modulation of muscle fiber structure and metabolic properties, the enhancement of antioxidant defense systems, and the regulation of gut microbiota. By consolidating findings from these diverse nutritional strategies, this review aims to provide a structured reference for researchers and feed formulators seeking to improve poultry meat quality through precision nutrition.

## 2. Literature Review Methodology

This narrative review was conducted following a structured literature search and screening process informed by the PRISMA guidelines. A systematic literature search was conducted in PubMed, Web of Science, and Google Scholar from January 2000 to March 2026. The search strategy centered on core keywords including “meat quality”, “poultry”, “amino acids”, “fatty acids”, “vitamins”, “minerals”, “probiotics”, and “plant extracts”. Studies were included if they were original research or peer-reviewed reviews published in English, evaluated at least one meat quality parameter (color, pH, water-holding capacity, tenderness, or flavor-related compounds), involved nutritional interventions in poultry species, and provided quantitative data. Studies were excluded if they focused exclusively on growth performance without meat quality assessment, used in vitro models without in vivo validation, were conference abstracts or theses, lacked a control group, or were duplicate publications.

The initial search yielded 420 records. After removing duplicates (*n* = 82), 338 records were screened by title and abstract, of which 124 were excluded as irrelevant. The remaining 214 full-text articles were assessed for eligibility, and 153 were excluded due to lack of quantitative data (*n* = 48), inappropriate intervention or species (*n* = 52), insufficient methodological detail (*n* = 36), or duplicate publication (*n* = 17). Finally, 61 articles were included in this review. Key information extracted from each study included poultry species, nutritional intervention, meat quality parameters, main findings, and proposed mechanisms. Data were synthesized narratively by nutrient category around three recurring mechanistic themes: modulation of muscle fiber structure, enhancement of antioxidant defense, and regulation of gut microbiota (gut–muscle axis).

## 3. Indicators for Evaluating Meat Quality

Poultry meat quality, as a composite trait, serves as the core criterion for evaluating its commercial value, sensory acceptability, and nutritional worth. It is constituted by a series of interrelated physical indicators, chemical parameters, and flavor characteristics. These metrics not only influence consumers’ immediate purchasing decisions but also determine subsequent processing, storage, and consumption rate. A systematic exposition of the principal dimensions for assessing meat quality lays the foundation for understanding the mechanisms of action and evaluating the efficacy of subsequent nutritional regulation strategies.

### 3.1. Physical Specifications

Physical indicators are traits directly measurable through sensory evaluation or instruments, forming the foundation for determining meat appearance, texture, and processing characteristics. The physical quality of poultry meat is primarily defined by four key indicators that collectively establish the product’s commercial appearance and eating experience. Meat color, as the primary visual signal, depends on myoglobin status and can be quantified by lightness L*, redness a*, and yellowness b*. It directly influences consumer purchasing decisions [10]. pH reflects the degree of post-slaughter muscle glycolysis, where both its rate of decline and final value are critical. Excessively rapid or slow changes can lead to pale color and poor texture, resulting in PSE (pale, soft, exudative) or DFD (dark, firm, dry) meat [11]. Water-holding capacity (water retention) indicates the muscle’s ability to retain moisture, assessed through drip loss and cooking loss. This metric not only affects meat juiciness and tenderness but also directly relates to economic yield [12]. Tenderness, central to edible quality, is typically objectively measured by shear force values and is jointly regulated by muscle fiber characteristics, connective tissue content, and post-slaughter aging processes [13]. These interrelated physical attributes form the fundamental framework for the perceived quality of meat products.

### 3.2. Chemical Indicators

The chemical indicators of poultry meat primarily include moisture, protein, and intramuscular fat. Its nutritional quality and flavor potential are jointly determined by chemical composition and microstructure. As the most abundant component, moisture distribution and binding forms directly regulate meat water-holding capacity, juiciness, and tenderness thresholds [14]. Protein content and amino acid composition—particularly the balance of essential amino acids—form the foundation for assessing nutritional quality. Certain amino acids (e.g., glutamic acid, glycine) serve as key precursors for flavor development [15]. Moderate increases in intramuscular fat content significantly enhance meat tenderness, juiciness, and flavor. Its fatty acid composition—including the polyunsaturated/monounsaturated fatty acid ratio and n-6/n-3 ratio—directly influences nutritional value and oxidative stability [16]. At the microstructural level, muscle fiber type, diameter, and density form the structural foundation of meat tenderness. Collagen, as the primary component of connective tissue, exhibits key limiting factors for meat tenderness and the basis for regulating toughness through its total content, the ratio of Type I to Type III collagen, and cross-linking density [17]. These chemical and structural elements are interrelated, collectively forming the material foundation for the nutritional essence and flavor potential of poultry meat.

### 3.3. Formatting Flavor Characteristics

**Figure 1:**
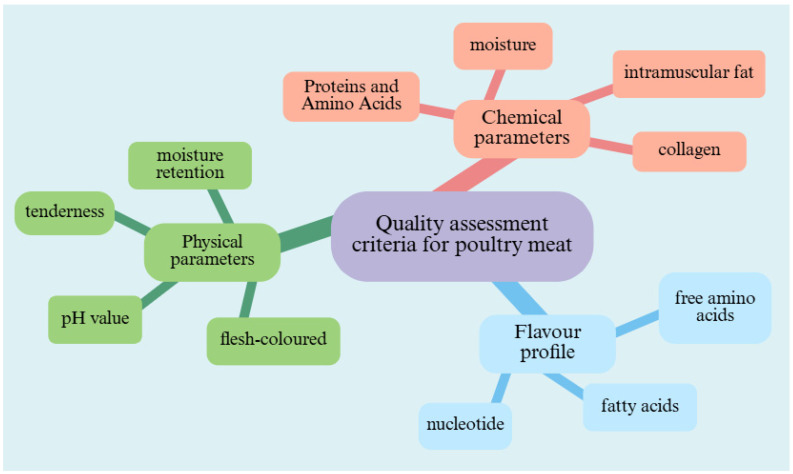
Classification of poultry meat quality evaluation indicators: indicator categories.

The formation of poultry meat’s unique flavor fundamentally depends on the composition, content, and interactive transformations during thermal processing of three key flavor precursors in muscle tissue: free amino acids, fatty acids, and nucleotides. Among amino acids, cysteine, glutamic acid, and glycine serve not only as fundamental protein building blocks but also as crucial substrates for the Maillard reaction during heating, yielding rich meat aroma and umami flavor [18]. The composition of fatty acids, particularly the content of polyunsaturated fatty acids (PUFAs), can generate volatile flavor compounds such as aldehydes, ketones, and alcohols through oxidative degradation pathways during thermal processing or storage, imparting characteristic lipid aromas to meat products [19]. Furthermore, inosine monophosphate (IMP), a key umami nucleotide, exhibits a positive correlation between its concentration and the perceived intensity of savory flavor in muscle tissue. It is a core factor influencing the sensation of “aftertaste” and the overall richness of flavor [20]. The content, proportions, and interactions of these compounds during processing collectively form the material basis for the flavor characteristics of poultry meat. The classification system for poultry meat quality evaluation indicators is shown in Figure 1.

The classification of meat quality indicators (physical specifications, chemical indicators, and flavor characteristics) is based on the integrated framework from previous studies [2,10,11,12,13,14,15,16,17,18,19,20].

## 4. Research on Nutritional Regulation in Poultry Meat Quality

### 4.1. Regulatory Role of Protein and Amino Acids

Proteins and amino acids influence poultry meat quality both as structural substrates that determine muscle fiber architecture and water-holding capacity, and as signaling molecules that regulate antioxidant defense, energy metabolism, and stress responses.

Within the framework of precision nutrition, moderately reducing crude protein levels in feed while supplementing essential amino acids has emerged as a sustainable production strategy that balances feeding efficiency, reduces nitrogen emissions, and potentially enhances meat quality. A consistent finding across multiple studies is that reducing crude protein from conventional levels (18–20%) to moderately lower levels (15–16%) while supplementing lysine and methionine improves meat tenderness and water-holding capacity. For instance, feeding slow-growing broilers a 16% crude protein diet with 0.99% lysine reduced muscle fiber diameter and shear force [4]. Similarly, in Taihe Black chickens, 15% crude protein with elevated metabolizable energy increased total amino acids, essential amino acids, and flavor nucleotides (IMP, AMP) in breast muscle [21]. However, a critical threshold exists: reducing crude protein below 15% without precise amino acid balancing triggers adverse effects including abnormal intramuscular fat accumulation and increased drip loss, ultimately compromising meat quality and production efficiency [22].Within this regulatory framework, methionine and lysine play pivotal roles as the primary and secondary limiting amino acids, respectively. Research indicates that supplementing diets with 0.50% methionine during the early rearing phase and 0.44% during the late phase enhances total antioxidant capacity in breast muscle, boosts glutathione peroxidase activity, and significantly reduces malondialdehyde content. These effects collectively improve muscle oxidative stability and water-holding capacity [23]. Further research indicates that incorporating 0.1% coated methionine into maternal diets, achieving a total methionine concentration of 0.37%, significantly improved offspring growth performance, breast and leg muscle weight, and reduced muscle tenderness shear force. Conversely, adding 0.2% to reach 0.47% total methionine produced adverse effects, decreasing offspring body weight and dressing percentage while causing quality fluctuations such as paler meat color and reduced pH [24]. Lysine improves water retention by increasing myosin solubility and slowing post-slaughter pH decline, significantly reducing drip and cooking loss while optimizing color stability [25]. Further studies confirm that progressively increasing digestible lysine levels in the diet during the finishing phase (23–38 days of age) linearly enhances broiler weight gain, improves feed conversion ratio, and significantly increases breast meat yield while reducing abdominal fat deposition and intramuscular fat content. Based on linear breakpoint model analysis, the optimized digestible lysine concentrations for feed conversion ratio and breast meat yield were 1.01% and 1.02% respectively, exceeding breed-recommended values. This indicates modern high-breast-yield broiler breeds exhibit more precise and stringent lysine requirements [26].

Unlike limiting amino acids, functional amino acids improve meat quality by activating specific molecular signaling pathways rather than through structural changes alone. These primarily include arginine, tryptophan, guanidinoacetic acid, and others. Research indicates that supplementing broiler diets with 0.2% L-arginine, combined with a vitamin C and choline complex, significantly enhances feed conversion efficiency and reduces the severity of woody breast in birds aged 42–49 days [27]. Furthermore, a 0.4% L-arginine inclusion markedly reduced Ca^2+^/Mg^2+^-ATPase activity within the actin-myosin complex and promoted actin-myosin dissociation into actin and myosin, thereby further improving meat tenderness [28]. Further research confirms that intra-embryonic injection of 0.6 mg L-arginine significantly increases relative breast muscle weight post-hatch in broilers by upregulating mTOR signaling pathway-related gene expression. This also enhances plasma total protein, thyroid hormone concentrations, and muscle amino acid concentrations, thereby improving early breast muscle development [29]. Thus, the same amino acid produces different benefits at different doses, and the choice of dose should align with the specific production goal. Tryptophan demonstrates that efficacy depends on environmental context. Under high-density stress, 0.78% tryptophan reduces drip loss via serotonin-mediated stress alleviation [30]. Under low-stress conditions, however, its effects are minimal. This finding has practical implications: functional amino acids should be targeted to specific production contexts rather than applied universally. Guanidinoacetic acid, as a direct precursor of creatine, can effectively enhance muscle phosphocreatine reserves through nutritional regulation. Recent research indicates that dietary supplementation with 0.05–0.10% guanidinoacetic acid upregulates skeletal muscle expression of peroxisome proliferator-activated receptor gamma coactivator 1α (PGC-1α), enhancing mitochondrial function and promoting biosynthesis. This facilitates the conversion of muscle fibers from fast-twitch to slow-twitch types, thereby improving meat quality at a metabolic level [31]. Studies indicate that supplementing broiler diets with 1041–1760 mg/kg β-alanine significantly increases carnosine content and expression of related synthase genes (CARNS1, SLC6A6) in breast muscle, improves meat color and tenderness, and enhances muscle antioxidant capacity—providing evidence for functional amino acid regulation in broiler production [32].

In summary, limiting amino acids improve meat quality through structural and antioxidant pathways with strict threshold effects, whereas functional amino acids act via specific signaling molecules such as nitric oxide, serotonin, PGC-1α, and carnosine in a context-dependent manner. An optimal strategy for protein and amino acid nutrition requires maintaining crude protein above 15% with precise lysine and methionine supplementation, selecting functional amino acid doses based on specific production goals and stress conditions, and recognizing that more is not always better—nonlinear responses are the rule, not the exception.

### 4.2. Regulatory Functions of Lipids and Fatty Acids

Lipids influence poultry meat quality through a fundamental trade-off: increasing unsaturated fatty acids improves nutritional value but compromises oxidative stability. As monogastric animals, poultry have limited capacity for de novo fatty acid synthesis, making muscle fatty acid composition highly responsive to dietary lipid sources.

The choice of dietary lipid source determines both the nutritional value and the oxidative stability of poultry meat. Flaxseed oil, rich in n-3 polyunsaturated fatty acids (PUFAs), effectively increases alpha-linolenic acid (ALA), EPA, and DHA in chicken meat while reducing the n-6/n-3 ratio to recommended healthy ranges. However, this nutritional benefit comes at a cost: n-3 PUFA enrichment heightens lipid oxidation risk, necessitating concurrent antioxidant supplementation such as vitamin E to maintain meat quality and prevent rancidity [1]. Blending different lipid sources offers a practical compromise. Unlike soybean oil, which is high in n-6 PUFA and accelerates oxidation, rapeseed oil provides superior stability and juiciness due to its high oleic acid content. Combining linseed oil with rapeseed oil thus improves fatty acid profiles while preserving oxidative stability and sensory characteristics [29]. Further research indicates synergistic effects from lipid source combinations. Comparing the effects of soybean oil, goose fat, and their blended lipids on Landrace geese revealed that adding 10 g/kg soybean oil blended with 15 g/kg goose fat promoted small intestinal development, increased villus height-to-crypt depth ratio, enhanced amino acid retention and metabolism, and comprehensively improved growth performance and meat quality [33]. Additional studies confirmed that feeding tuna oil or its lipid-based nanoparticle form effectively elevated n-3 polyunsaturated fatty acid content in chicken meat while improving fatty acid composition, without adversely affecting growth performance, carcass traits, or meat quality. Although the enrichment efficiency in the nanoparticle group was slightly lower than that in the direct addition group due to processing heat loss, it still regulated blood parameters and maintained body health, indicating the potential of this technology for producing functional poultry meat [34]. Further research confirms that supplementing diets with 300 mg/kg medium-chain triglycerides significantly increases late-phase feed intake, body weight, and carcass yield in broilers. It also enhances leg and breast muscle production, reduces drip loss and pH decline, and elevates muscle saturated fatty acids, flavor-enhancing amino acids, and total amino acid content. This comprehensively improves broiler production performance and meat quality [35].

Functional fatty acids, particularly conjugated linoleic acid (CLA), have been progressively explored from phenotypic effects to molecular mechanisms. CLA possesses a unique “fat redistribution” effect: it reduces body fat percentage while increasing intramuscular fat (IMF) deposition, thereby enhancing meat tenderness, juiciness, and flavor [36]. Mechanistically, CLA activates peroxisome proliferator-activated receptors (PPARα and PPARγ), which respectively promote fatty acid β-oxidation and lipid synthesis, facilitating IMF deposition [37]. However, CLA supplementation may also be associated with meat color hardening and increased lipid oxidation risks. In practice, antioxidants such as vitamin E must be incorporated to maintain product stability [38]. Beyond CLA, other nutritional factors influence meat quality by precisely regulating key lipid metabolism genes. Research indicates that glycerol butyrate downregulates fatty acid synthase (FASN) expression in liver and adipose tissue, thereby reducing excessive abdominal fat deposition [39]. Further research indicates that dietary energy levels significantly modulate the expression and activity of lipoprotein lipase (LPL), thereby influencing tissue uptake of blood triglycerides. Gender dimorphism exists in the expression of key lipid metabolism genes, suggesting that precision nutrition strategies must account for the impact of sex factors on meat deposition efficiency [40].

In summary, lipid nutrition for meat quality is inherently a multi-objective optimization problem. The optimal strategy combines PUFA-rich sources for nutritional enhancement, MUFA-rich sources for oxidative stability, functional fatty acids for targeted fat redistribution, and proportional antioxidant supplementation calibrated to PUFA content. Dose–response relationships are non-linear across all categories, and sex-specific differences indicate that a one-size-fits-all approach is unlikely to succeed.

### 4.3. Regulatory Functions of Vitamins and Minerals

Vitamins and minerals influence poultry meat quality through an integrated antioxidant network. Vitamin E scavenges peroxyl radicals in cell membranes, vitamin C regenerates oxidized vitamin E in aqueous compartments, and selenium (as part of glutathione peroxidase) catalyzes the reduction of hydrogen peroxide and lipid peroxides. Together, these micronutrients maintain cellular structural integrity, support enzymatic reactions, and mitigate oxidative stress—a particular concern for modern meat poultry subjected to accelerated growth rates.

#### 4.3.1. Synergistic Effects of the Vitamin Antioxidant Network

Vitamin E (α-tocopherol), acting as a lipid-soluble chain-breaking antioxidant in cell membranes, effectively maintains the structural and functional integrity of cell membranes by scavenging peroxy radicals in lipid peroxidation chain reactions [41]. Research indicates that dietary supplementation with vitamin E (200 IU/kg) under heat stress conditions significantly increases α-tocopherol concentrations in broiler breast muscle, enhances muscle deposition, and reduces post-slaughter drip loss. Simultaneously effectively delaying myoglobin oxidation to metmyoglobin, preserving bright red meat color and extending shelf life. By inhibiting the formation of malondialdehyde (MDA), a lipid oxidation product, it systematically enhances meat oxidative stability [42]. Further research indicates that supplementing with 200–250 mg/kg vitamin C under heat stress conditions, combined with an early fasting strategy, significantly reduces serum corticosterone levels in broilers, upregulates hepatic antioxidant enzyme expression, and mitigates adverse effects of stress on growth performance and meat quality [43]. Moreover, beyond regulating calcium-phosphorus metabolism, vitamin D_3_ enhances meat tenderness by elevating muscle calcium ion concentrations, activating the calpain system, and promoting post-slaughter myofibrillar skeletal protein degradation [44]. This distinct pathway illustrates that vitamins can improve meat quality through multiple, non-overlapping mechanisms.

#### 4.3.2. Advantages of Bioavailability in Organic Trace Elements

Traditional inorganic minerals face limitations in feed applications due to their low bioavailability and susceptibility to ion antagonism. In contrast, organic trace elements offer a novel approach to enhancing poultry meat quality through superior absorption efficiency and biological activity. Among these, selenomethionine (SeMet) can be actively absorbed via the methionine transport pathway and non-specifically substituted for methionine in muscle protein synthesis. This significantly enhances glutathione peroxidase (GSH-Px) activity, thereby scavenging hydrogen peroxide and lipid peroxides. Consequently, it reduces drip loss and cooking loss while improving meat water-holding capacity [45]. At equivalent addition concentrations of 0.5 mg/kg, organic selenium sources such as selenium-enriched yeast (SY) and selenomethionine (SM) demonstrated superior deposition efficiency in chicken tissues and serum compared to sodium selenite (SS). Notably, selenium-enriched yeast significantly elevated selenium content in breast and leg muscles while effectively enhancing serum total antioxidant capacity, thereby more efficiently improving meat quality by indirectly boosting the body’s antioxidant status [46]. Zinc contributes through membrane integrity. Organic zinc elevates zinc content in breast and leg muscles while promoting selenium and α-tocopherol deposition within tissues [47]. Further research indicates organic zinc maintains structural integrity of epithelial and muscle cell membranes, reducing juice exudation caused by increased membrane permeability [48]. As a key component of the Glucose Tolerance Factor (GTF), organic chromium enhances insulin sensitivity and promotes tissue glucose uptake. Under heat stress conditions, organic chromium supplementation improves energy metabolism, reduces serum corticosterone concentrations, thereby mitigating adverse effects of stress on meat quality and enhancing slaughter performance [49].

In summary, vitamins and minerals form an integrated network in which each component plays a distinct role: vitamin E provides membrane protection, vitamin C enables recycling, vitamin D_3_ activates tenderness-related proteases, and organic trace elements offer superior bioavailability through active transport. The optimal strategy requires maintaining vitamin E at approximately 200 IU/kg, supplementing vitamin C under stress conditions, and replacing inorganic trace minerals with organic forms to achieve 2–3 times higher tissue deposition. However, the optimal ratios among these micronutrients remain undefined, and most studies examine single nutrients rather than their synergistic interactions.

### 4.4. Regulatory Effects of Plants and Plant Extracts

Plants and their extracts are rich in bioactive compounds including polyphenols, flavonoids, saponins, polysaccharides, and essential oils. Unlike isolated nutrients, these substances act through multiple mechanisms simultaneously, offering both nutritional and pharmacological functions with reduced risk of drug resistance and residue concerns [8]. The primary mechanisms include Nrf2-mediated activation of endogenous antioxidant defenses, gut microbiota modulation, and direct anti-inflammatory effects.

Polyphenols, widely present in grape seeds, tea leaves, mulberry leaves, and onions, extend beyond direct free radical scavenging to systematically regulate cellular signaling pathways. Their primary mechanism involves disrupting the Nrf2-Keap1 complex, promoting nuclear translocation of the transcription factor Nrf2, and upregulating multiple antioxidant-related genes including phase II detoxification enzymes [50]. This mechanistic distinction from direct-acting antioxidants such as vitamin E has important practical implications: Nrf2 activation produces longer-lasting effects due to sustained upregulation of antioxidant enzymes, whereas vitamin E provides rapid but transient protection. In broiler production, supplementing polyphenol complexes such as grape seed extract significantly reduces the incidence and severity of woody breast through decreased muscle fiber diameter and increased fiber density, while also inhibiting oxidation of unsaturated fatty acids, optimizing fatty acid composition, and enhancing n-3 polyunsaturated fatty acid deposition rates [42]. Plant essential oils such as thymol, carvacrol, and tea tree oil exhibit potent antibacterial activity by disrupting pathogen cell membrane structures, thereby optimizing gut microbiota composition, reducing systemic inflammation and endotoxemia, and enabling the body to allocate more nutritional resources toward muscle growth and repair. Combining tea tree oil with probiotics further reduces drip loss in broiler breast meat, likely through inhibiting inflammatory factor release and protecting cell membrane integrity [51].

Beyond these common plant-based ingredients, numerous other natural additives demonstrate significant meat quality improvement effects. Studies confirm that adding 0.02% rosemary extract (RE) or 15% rosemary residue (RR) to diets significantly reduces shear force and crude fat content in goose meat, increases essential amino acid and polyunsaturated fatty acid ratios, and enhances muscle antioxidant capacity. The RE also effectively inhibits microbial growth and lipid oxidation during storage [52]. Betaine reduces drip loss and malondialdehyde concentrations in duck meat by enhancing muscle antioxidant activity and regulating myogenesis-related gene expression, while also increasing certain amino acid contents [53]. Neomarcella leaf extract significantly improves broiler breast meat pH, shear force, and water-holding capacity by activating the p38 MAPK/Nrf2/ARE signaling pathway to enhance muscle antioxidant enzyme activity [54]. Purslane extract effectively reduces drip loss, cooking loss, and shear force in Wenchang chickens by regulating gut microbiota and enriching tryptophan, arginine, and proline metabolic pathways, while simultaneously increasing umami amino acids and inosine monophosphate content [55]. Additionally, the combination of moringa leaf extract and zinc nanoparticles has been shown to lower blood lipids and enhance muscle antioxidant capacity [56].

In summary, some plant extracts provide effective and safe natural solutions for healthy poultry farming by enhancing meat quality, boosting antioxidant capacity through Nrf2 activation, and regulating gut microbiota. However, interactions between plant extracts and other nutritional strategies remain largely unexplored and warrant further investigation.

### 4.5. Regulatory Effects of Probiotics

Probiotics, as a class of active microorganisms beneficial to host health, exert their primary effects through mechanisms including competitive exclusion to inhibit pathogen colonization in the gut, promoting mucin secretion by intestinal goblet cells to enhance the physical barrier function, and stimulating the host to produce antimicrobial peptides such as defensins to establish a chemical barrier [57]. Furthermore, short-chain fatty acids produced by gut microbiota fermentation not only supply energy to intestinal epithelial cells but also enter the bloodstream to regulate lipid metabolism and insulin sensitivity, thereby indirectly improving carcass composition and reducing abdominal fat deposition [8].

Regarding meat quality regulation, multiple studies have revealed the effects of probiotics and their synergistic components: Research indicates that supplementing diets with *Lactobacillus rhamnosus* (LGG) and xylooligosaccharides (XOS) increases polyunsaturated fatty acid (PUFA) content in leg muscles while improving meat nutritional value and gut microbiota by enhancing the abundance of *Lactobacillus* and *Bifidobacterium* genera [58]. Addition of *Clostridium butyricum* significantly enhances growth performance and breast meat quality in Beijing ducks while increasing the composition of PUFAs and flavor-enhancing amino acids, a mechanism linked to regulating lipid metabolism and boosting muscle antioxidant capacity [59]. *Lactobacillus plantarum* regulates duck meat flavor via the “gut–muscle axis,” manifested by elevated linoleic acid content and characteristic volatile compounds such as valeraldehyde, hexanal, and 2,3-octanedione. Metagenomic and transcriptomic analyses further confirm this process involves fatty acid metabolism genes including acetyl-CoA carboxylase β (ACACB), acetyl-CoA acetyltransferase 2 (ACAT2), aldehyde dehydrogenase 1 family member A1 (ALDH1A1), and alterations in amino acid metabolic pathways [60]. Similarly, *Bacillus laterosporus* enhances α-linolenic acid content and reduces the trans-oleic acid to arachidonic acid ratio by modulating broiler cecal microbiota and their metabolites. This potentially optimizes meat flavor and nutritional value while boosting antioxidant capacity, without significantly affecting shear force, pH, or color parameters of meat color [61].

A critical pattern emerges across these studies: probiotics demonstrate greater efficacy under suboptimal rearing conditions—such as high density or heat stress—than under ideal conditions. This suggests that probiotics primarily mitigate environmental stressors rather than enhance baseline quality. Furthermore, effects are strain-specific; beneficial outcomes documented for one strain cannot be generalized to uncharacterized probiotics. Most studies examine single strains, leaving synergistic effects of multi-strain combinations unexplored. In summary, probiotics effectively improve the fatty acid composition, flavor compound accumulation, and nutritional value of poultry meat by regulating the gut microbiota, host metabolism, and antioxidant status through multiple pathways, providing crucial technical support for green and healthy animal husbandry. The regulatory mechanisms of key nutrients on poultry meat quality are shown in Table 1.

## 5. Conclusions

Poultry nutrition management has undergone a strategic shift from growth-centered to quality-focused production. This review demonstrates that nutritional regulation improves poultry meat quality through three core mechanisms: modulation of muscle fiber structure, enhancement of antioxidant defense systems, and regulation of the gut–muscle axis.

At the protein and amino acid level, moderate crude protein reduction (15–16%) with precise lysine and methionine supplementation optimizes muscle fiber architecture and water-holding capacity. Functional amino acids such as arginine, tryptophan, and guanidinoacetic acid act through specific signaling pathways to reduce woody breast, alleviate stress, and promote slow-twitch fiber conversion. For lipids, n-3 polyunsaturated fatty acid enrichment improves nutritional value but requires concurrent vitamin E supplementation to maintain oxidative stability, while conjugated linoleic acid increases intramuscular fat deposition via PPAR pathway activation. At the micronutrient level, an integrated antioxidant network comprising vitamin E, vitamin C, and organic selenium alleviates oxidative stress and reduces meat defects such as PSE and woody breast. Plant extracts activate the Nrf2 pathway to enhance endogenous antioxidant capacity, and probiotics improve meat quality indirectly through gut microbiota modulation and the gut–muscle axis.

The shift from quantity to quality is increasingly reflected in today‘s commercial breeds. Modern high-yield strains exhibit greater susceptibility to quality defects such as woody breast and white striping compared to strains from the 2000s, underscoring the need for nutritional strategies that mitigate stress-induced myopathies. However, direct comparative data on meat flavor, tenderness, and nutritional composition between poultry from these two decades remain limited. Future research should compare meat quality attributes between contemporary and legacy strains to quantify the impact of genetic selection and validate the applicability of historical nutritional data to modern production systems. Additionally, multi-omics approaches are needed to map molecular networks linking nutrients to meat quality traits, and dynamic precision feeding systems should be developed for commercial application.

## Figures and Tables

**Table 1:** The effect of nutritional management strategies on poultry meat quality.

Nutritional Category	Subcategory	Main Mechanisms of Action	Main Effects on Meat Quality	References
Proteins and Amino Acids	Total Protein and Restrictive Amino Acids	Optimizing myofiber structure; enhancing antioxidant capacity; regulating energy metabolism	Reducing shear force; increasing umami nucleotides; improving WHC and color stability	[4,21,22,23,24,25,26]
	Functional Amino Acids and Their Derivatives	Promoting fiber type transformation; enhancing mitochondrial function; alleviating stress	Reducing woody breast; improving tenderness; reducing drip loss; enhancing antioxidant capacity	[27,28,29,30,31,32]
Lipids and Fatty Acids	Source of Oil and Fatty Acid Profile	Modulating fatty acid composition; optimizing n-6/n-3 ratio	Increasing EPA/DHA and flavor amino acids; reducing drip loss; improving flavor and juiciness	[1,29,33,34,35]
	Functional fatty acids	Activating PPARα/γ pathways; regulating lipid metabolism genes	Increasing intramuscular fat deposition; improving tenderness and flavor; reducing abdominal fat	[36,37,38,39,40]
Vitamins and Minerals	Vitamin Antioxidant Network	Scavenging lipid peroxyl radicals; activating calpain system	Maintaining red color; reducing drip loss; inhibiting lipid oxidation; improving tenderness	[41,42,43,44]
	Organic trace minerals	Enhancing GSH-Px activity; maintaining membrane integrity; improving insulin sensitivity	Improving WHC; increasing Se/Zn deposition; enhancing T-AOC; alleviating stress	[45,46,47,48,49]
Plants and Plant Extracts	Polyphenols and Essential Oils	Activating Nrf2 pathway; scavenging free radicals; optimizing gut microbiota	Reducing woody breast; decreasing myofiber diameter; inhibiting lipid oxidation; reducing drip loss	[42,50,51]
	Other naturally extracted compounds	Modulating gut microbiota; regulating myogenesis-related genes	Reducing shear force; increasing umami amino acids and IMP content	[52,53,54,55,56]
Probiotics	Probiotics and Their Synergists	Enhancing intestinal barrier; regulating gut–muscle axis and lipid metabolism genes	Increasing PUFA and flavor amino acids; improving fatty acid composition; modulating volatile flavor compounds	[8,57,58,59,60,61]

## Data Availability

No new data were created or analyzed in this study. Data sharing is not applicable to this article.
